# Supplementary material for: Gaussian-Schell analysis of the transverse spatial properties of high-harmonic beams
Source: Sci Rep. 2016 Jul 28;6:30504. doi: 10.1038/srep30504 (PMC4964618; doi:10.1038/srep30504)
Supplement: Supplementary Information [file srep30504-s1.pdf]

# Gaussian-Schell analysis of the transverse spatial properties of high-harmonic beams: Supplementary Information

**David T. Lloyd[1*], Kevin O'Keeffe[1,2], Patrick N. Anderson[1], and Simon M. Hooker[1]**

[1]Department of Physics, University of Oxford, Clarendon Laboratory, Parks Road, Oxford OX1 3PU, UK
[2]Department of Physics, Swansea University, Singleton Park, Swansea, SA2 8PP, UK
[*]Corresponding author: david.lloyd@physics.ox.ac.uk

## ABSTRACT

This document provides supplementary information to "Gaussian-Schell analysis of the transverse spatial properties of high-harmonic beams". A derivation of an expression for the complex coherence factor of a 1-D source of high order harmonics, presented in abbreviated form in the main text, is provided here with additional detail.

## 1 Theory of High Harmonic Spatial Coherence

We write the electric field of harmonic order $q$ as:

$$E_q(x_i,t) = A_q \varepsilon_q(x_i)\chi_q(t)\exp[\mathrm{i}\phi_q(x_i,t)] \tag{1}$$

where $\varepsilon_q$ and $\chi_q$ are real, positive functions corresponding to envelopes in space and time, respectively, of the electric field, the subscript $q$ links the quantity explicitly with harmonic order $q$ and $x_i$ refers to transverse position in a plane where the harmonic is generated. Here, by using separable functions for the spatial and temporal parts of the harmonic field envelope, we have implicitly assumed no space-time coupling (STC) is present in the harmonic amplitude. Furthermore we concern ourselves with a one-dimensional harmonic source: it extends in the x-direction only.

The complex coherence factor parameterises the spatial coherence of the field. It can be written as:

$$\mu_q = \frac{\langle E_q(x_1,t)E_q(x_2,t)^*\rangle}{\sqrt{\langle |E_q(x_1,t)|^2\rangle\langle |E_q(x_2,t)|^2\rangle}} \tag{2}$$

where the angle brackets denote a time average. Substituting Eq.1 into 2:

$$\begin{aligned}\mu_q = \frac{A_q^2\varepsilon_q(x_1)\varepsilon_q(x_2)\langle\chi_q(t)^2\exp\{i[\phi_q(x_1,t)-\phi_q(x_2,t)]\}\rangle}{A_q^2\varepsilon_q(x_1)\varepsilon_q(x_2)\langle\chi_q(t)^2\rangle} \\ = \frac{\langle\chi_q(t)^2\exp\{i[\phi_q(x_1,t)-\phi_q(x_2,t)]\}\rangle}{\langle\chi_q(t)^2\rangle}\end{aligned} \tag{3}$$

hence

$$|\mu_q| = \left|\frac{\langle\chi_q(t)^2\exp\{\mathrm{i}\Delta\phi_q\}\rangle}{\langle\chi_q(t)^2\rangle}\right| \tag{4}$$

where $\Delta\phi_q = \phi_q(x_1,t) - \phi_q(x_2,t)$.

### 1.1 Taylor Expansion of Phase Difference

If the difference between the phases $\phi_q(x_1)$ and $\phi_q(x_2)$ is sufficiently small, a Taylor expansion may be used:

$$\exp(\mathrm{i}\Delta\phi_q) = 1 + \mathrm{i}\Delta\phi - \frac{\Delta\phi^2}{2}\ldots \tag{5}$$

substituting into equation 4 and expanding out:

$$|\mu_q| \approx \left|\frac{\langle\chi_q(t)^2\left(1+\mathrm{i}\Delta\phi_q-\frac{\Delta\phi^2}{2}\right)\rangle}{\langle\chi_q(t)^2\rangle}\right| = \left[1+\frac{\langle\chi_q(t)^2\Delta\phi_q\rangle^2}{\langle\chi_q(t)^2\rangle^2}+\frac{\langle\chi_q(t)^2\Delta\phi_q^2\rangle^2}{4\langle\chi_q(t)^2\rangle^2}-\frac{\langle\chi_q(t)^2\Delta\phi_q^2\rangle}{2\langle\chi_q(t)^2\rangle}-\frac{\langle\chi_q(t)^2\Delta\phi_q^2\rangle}{2\langle\chi_q(t)^2\rangle}\right]^{\frac{1}{2}}. \quad (6)$$

Disregarding the higher order term proportional to $\langle\chi_q(t)^2\Delta\phi_q^2\rangle^2$ and applying a binomial expansion of the square root yields:

$$|\mu_q| \approx 1-\frac{1}{2}\left(\frac{\langle\chi_q(t)^2\Delta\phi_q^2\rangle}{\langle\chi_q(t)^2\rangle}-\frac{\langle\chi_q(t)^2\Delta\phi_q\rangle^2}{\langle\chi_q(t)^2\rangle^2}\right) \quad (7)$$

where the second term in equation 7 resembles the variance of $\Delta\phi_q$ *modulated (or windowed) by the temporal envelope of the harmonic pulse*.

### 1.2 Full Expression for the Harmonic CCF

The harmonic phase can be written as $\phi_q = q\phi_0(x,t) - \alpha_j^q I_0(x,t)$, where $\phi_0$ and $I_0$ are the phase and intensity of the fundamental beam, respectively and $\alpha_j^q$ is an order dependent parameter related to the trajectory associated with the harmonic emission. The phase difference $\Delta\phi_q$ is then:

$$\begin{aligned}\Delta\phi_q = q[\phi_0(x_1,t)-\phi_0(x_2,t)] - \alpha_j^q[I_0(x_1,t)-I_0(x_2,t)]\\ = q\Delta\phi_0 - \alpha_j^q\Delta I_0\end{aligned} \quad (8)$$

where $\Delta I_0 = I_0(x_1,t) - I_0(x_2,t)$. Substituting into equation 7 yields:

$$|\mu_q| \approx 1-\frac{1}{2}q^2\left[\frac{\langle\chi_q^2\Delta\phi_0^2\rangle}{\langle\chi_q^2\rangle}-\frac{\langle\chi_q^2\Delta\phi_0\rangle^2}{\langle\chi_q^2\rangle^2}\right]-\frac{1}{2}(\alpha_j^q)^2\left[\frac{\langle\chi_q^2\Delta I_0^2\rangle}{\langle\chi_q^2\rangle}-\frac{\langle\chi_q^2\Delta I_0\rangle^2}{\langle\chi_q^2\rangle^2}\right]-\frac{\langle\chi_q^2 q\Delta\phi_0\alpha_j^q\Delta I_0\rangle}{\langle\chi_q^2\rangle}+\frac{\langle\chi_q^2 q\Delta\phi_0\rangle\langle\chi_q^2\alpha_j^q\Delta I_0\rangle}{\langle\chi_q^2\rangle^2}. \quad (9)$$

Eq. 9 can be expressed in a more compact form as:

$$|\mu_q| \approx 1-\frac{1}{2}\left[q^2V_q'(\Delta\phi_0)+(\alpha_j^q)^2V_q'(\Delta I_0)+2q\alpha_j^qC_q'(\Delta\phi_0,\Delta I_0)\right] \quad (10)$$

where

$$V_q'(F) = \frac{\langle\chi_q^2(F)^2\rangle}{\langle\chi_q^2\rangle}-\frac{\langle\chi_q^2F\rangle^2}{\langle\chi_q^2\rangle^2} \quad (11)$$

and

$$C_q'(F,G) = \frac{\langle\chi_q^2FG\rangle}{\langle\chi_q^2\rangle}-\frac{\langle\chi_q^2F\rangle\langle\chi_q^2G\rangle}{\langle\chi_q^2\rangle^2} \quad (12)$$

can be thought of as the variance and covariance functions, respectively, weighted by the harmonic temporal profile $\chi_q^2$. If, over the duration of the harmonic emission, the variation in the driver phase difference ($\Delta\phi_0$) is 'statistically independent' of the variation in the driver intensity difference ($\Delta I_0$), or if either $\Delta\phi_0$ or $\Delta I_0$ are independent of time or zero, then $C_q'(\Delta\phi_0,\Delta I_0)=0$.

Eq 7 can be modified to yield an expression for the driver CCF evaluated *during the emission of harmonic q*:

$$|\mu_0'| \approx 1-\frac{1}{2}\left(\frac{\langle\chi_q(t)^2\Delta\phi_0^2\rangle}{\langle\chi_q(t)^2\rangle}-\frac{\langle\chi_q(t)^2\Delta\phi_0\rangle^2}{\langle\chi_q(t)^2\rangle^2}\right). \quad (13)$$

This CCF likely differs to the true CCF of the driver (i.e. $|\mu_0|$) which is evaluated over the entire duration of the driver pulse. Using equation 13, we can write the harmonic CCF as:

$$|\mu_q| \approx 1-q^2(1-|\mu_0'|)-\frac{1}{2}(\alpha_j^q)^2V_q'(\Delta I_0)-q\alpha_j^qC_q'(\Delta\phi_0,\Delta I_0). \quad (14)$$

Writing the intensity difference as $\Delta I_0 = I_{00}\chi_0^2[\varepsilon(x_1)^2-\varepsilon(x_2)^2]$, where $I_{00}$ is the peak *driver* intensity, the harmonic CCF becomes:

$$|\mu_q| \approx 1-q^2(1-|\mu_0'|)-\frac{1}{2}\alpha^2I_{00}^2[\varepsilon(x_1)^2-\varepsilon(x_2)^2]^2V_q'(\chi_0^2)-q\alpha_j^qI_{00}[\varepsilon(x_1)^2-\varepsilon(x_2)^2]C_q'(\Delta\phi_0,\chi_0^2) \quad (15)$$
